# UAV multispectral multi-domain feature optimization for the air-to-ground recognition of outdoor injured human targets under cross-scene environment

**DOI:** 10.3389/fpubh.2023.999378

**Published:** 2023-02-09

**Authors:** Fugui Qi, Juanjuan Xia, Mingming Zhu, Yu Jing, Linyuan Zhang, Zhao Li, Jianqi Wang, Guohua Lu

**Affiliations:** ^1^Department of Military Biomedical Engineering, Fourth Military Medical University, Xi'an, China; ^2^Drug and Instrument Supervisory and Test Station of Xining Joint Service Support Center, Lanzhou, China

**Keywords:** UAV, multispectral detection, human target detection, air-to-ground recognition, cross-scene multi-domain feature joint optimization

## Abstract

**Objective:**

UAV-based multispectral detection and identification technology for ground injured human targets, is a novel and promising unmanned technology for public health and safety IoT applications, such as outdoor lost injured searching and battlefield casualty searching, and our previous research has demonstrated its feasibility. However, in practical applications, the searched human target always exhibits low target-background contrast relative to the vast and diverse surrounding environment, and the ground environment also shifts randomly during the UAV cruise process. These two key factors make it difficult to achieve highly robust, stable, and accurate recognition performance under the cross-scene situation.

**Methods:**

This paper proposes a cross-scene multi-domain feature joint optimization (CMFJO) for cross-scene outdoor static human target recognition.

**Results:**

In the experiments, we first investigated the impact severity of the cross-scene problem and the necessity to solve it by designing 3 typical single-scene experiments. Experimental results show that although a single-scene model holds good recognition capability for its scenes (96.35% in desert scenes, 99.81% in woodland scenes, and 97.39% in urban scenes), its recognition performance for other scenes deteriorates sharply (below 75% overall) after scene changes. On the other hand, the proposed CMFJO method was also validated using the same cross-scene feature dataset. The recognition results for both individual scene and composite scene show that this method could achieve an average classification accuracy of 92.55% under cross-scene situation.

**Discussion:**

This study first tried to construct an excellent cross-scene recognition model for the human target recognition, named CMFJO method, which is based on multispectral multi-domain feature vectors with scenario-independent, stable and efficient target recognition capability. It will significantly improve the accuracy and usability of UAV-based multispectral technology method for outdoor injured human target search in practical applications and provide a powerful supporting technology for public safety and health.

## 1. Introduction

Injured or trapped human searching in outdoor environments after natural disasters or outdoor accidents come to be important threats to public safety with the prevalence of outdoor sports and frequent occurrence of natural disasters in recent years, it also poses higher challenges to public security and health technology. Outdoor injured human searching in public security field mainly includes two categories (1): (1) One is about the trapped survivor detection under ruins in the abnormal post-disaster environment (earthquakes, building collapses, landslides, etc). To address this problem, our group firstly proposed the *bio-radar* detection technology (2) in the field of Disaster Rescue Medicine and developed a series of bio-radar equipment, which could acquire survivor's vital sign (3–6), locations (7) and even behaviors (8, 9) through ruins or wall. Based on the IoT technology, our bio-radar equipment radar equipment and other equipment together form the land-based search-rescue IoT equipment system, which has been successfully applied in many post-disaster search-rescue operations.

Another widespread and frequently occurring scenario is about the injured person searching in normal outdoor environment, namely a vast and diverse natural environment, like the lost hiker, emergency skydiving pilot, and even the wounded soldier after a field battle. Taking a lamentable and sensational sport accident for example, a large number of athletes suffered a safety accident due to the sudden change of weather in the 2021 4th Yellow River Shilin Mountain Marathon 100 km cross-country race in China (10). Many athletes experienced the severe Hypothermia phenomenon and were trapped in the mountains. Unfortunately, due to the lack of air-to-ground rapid search and location technology in the outdoor environment, information of the trapped people could not be sent back to the rear security center in time, resulting in a number of deaths that could not be treated in time. Therefore, it is necessary to develop an unmanned rapid intelligent search-rescue IoT technology, so as to realize the automatic air-to-ground detection and identification of ground injured human targets and automatically transmitting information to the rear command center in time, and finally providing efficient and novel technical support for outdoor injured human search-rescue operation.

In this paper, we propose an automatic air-to-ground recognition technology of outdoor ground injured human target based on the UAV-based multispectral system. Specifically, a novel Cross-scene Multi-domain Feature Joint Optimization (CMFJO) method based on the multispectral multi-domain features from multiple environmental scenes is first proposed, which can not only improve target recognition in a complex environment but also promote the recognition robustness to dynamically changing scenes for practical application.

## 2. Related work

This part refers to certain papers investigating the existing search-rescue technologies for outdoor injured human target, and discussing the pros and cons. For the above-mentioned severe mountain-forest outdoor environment, the conventional constrained method of pre-wearing auxiliary positioning device is not an ideal choice (like the portable radio station, wearable GPS personal terminal), which usually suffer from some inevitable deficiencies including increasing body load and vulnerability to extreme mountain-forest environments (11–13). Especially for soldiers, carrying equipment would easily expose themselves to the enemy. Currently, there are indeed some unconstrainedly unmanned aerial vehicle (UAV)-based air-to-ground detection technologies for human target search in ideal background environments, mainly relying on different carried detection payloads like RGB high-definition camera (14–16) or thermal imaging camera (17), no matter with a flat view (18–20) or top view (21). However, the RGB camera still appears insufficient resolution and low SNR when the detection distance is long or the object is similar in color to the environment, and even appears underexposure or overexposure when the ambient light changes (22). Similarly, the thermal signal of the human body would be covered by the halos when the ambient temperature is higher than 30°C.

As an optimized form of hyperspectral technology, UAV-based multispectral could relatively streamline data volume and realize real-time imaging processing by rationally selecting 4~10 characteristic spectrum bands for data processing (23), while ensuring a sufficient amount of information. By analyzing the differences in spectral characteristic curves between target and circumstances, specific features in different bands can be exploited to identify the target. Relying on this advantage, UAV-based multispectral detection technology is widely used in agricultural, forestry and environmental monitoring under low-altitude cruise conditions, such as the damage assessment to rapeseed crops after winter (24), decision support system design for variable rate irrigation (25), fast Xylella symptoms detection in olive trees (26), and inferring the spatial distribution of chlorophyll concentration and turbidity in surface waters to monitor the nearshore- offshore water quality (24).

However, for the rapid detection and identification of outdoor injured human subjects in an outdoor environment, the detection scenario is remarkably different from the above scenarios, showing more severe detection difficulties and challenges. Specifically, there is an unfavorable fact that the most common clothes of outdoor travelers and soldiers are camouflage clothes, which are too similar to the characteristics of the surrounding environment to distinguish. Moreover, the injured human target is just a tiny target with a much smaller size compared with the surrounding environment under an airborne view. Target to this challenge, only a few studies were carried out around the multispectral characteristics of camouflage and the identification of soldiers' camouflage equipment. For example, Wang et al. (27) explored the hyperspectral polarization characteristics of typical camouflage targets in desert background. Lagueux et al. (28–30) further measured the multispectral characteristics of the camouflage uniforms and some other soldier's camouflage equipment in different conditions, showing that the multispectral is promising to detect the camouflage equipment even under deliberate camouflage. Based on this advantage, PAR Government Systems Corporation even tried to detect and recognize the mine-like small target by adding a temporal dimension to the spectral processing (31) in desert background. In this regard, our group also tried to recognize the injured human targets in camouflage in a static outdoor environment based on UAV-multispectral features (32). In the latest research, researchers have even begun to study image target recognition under changing environmental factors (like illumination, seasonal and weather) based on deep learning (16, 33), but the object and the environmental background objects always keep consistent.

In general, research on the multispectral characteristics analysis and recognition of camouflage targets under a single static background has made gratifying progress. Unfortunately, in a practical air-to-ground searching operation, adverse ground environmental conditions will bring two critical challenges: (1) Complex outdoor ground environment components. During the detection of outdoor camouflaged injured human targets, any ground environment scene (desert, mountain forest or urban scene) is composed of many ground components, such as trees, green grass, yellow soil, stones and polychromatic plants, and their spectral characteristics even change with light and seasons. Compared to the ground environment, the injured human in camouflage clothes is just a small target and usually with similar camouflage color. Consequently, it's challenging to recognize the human target under such low target-ground contrast condition with high accuracy and robustness. (2) Cross ground environment or cross scene. For a practical outdoor urgent search mission, the ground environment is diverse and keeps switching dynamically and randomly. Consequently, traditional recognition methods and models trained based on multispectral characteristics from a specific single environment scene will perform poorly in an unknown scene, showing poor robustness and weak multi-scene stability.

## 3. UAV-based multispectral detection system

### 3.1. UAV-carrying system and ground workstation

**Figure 1:**
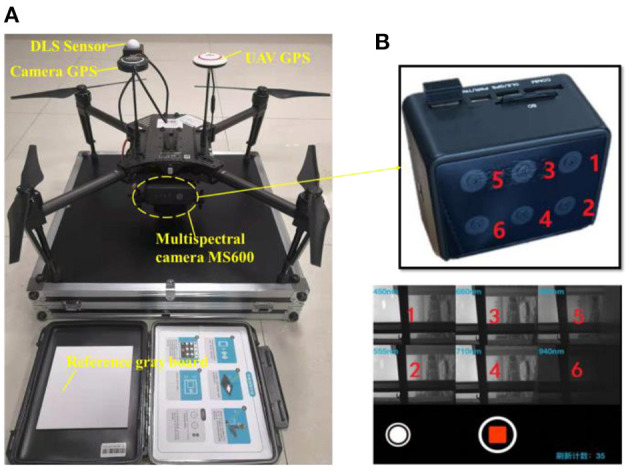
**(A)** The overall architecture of the UAV multispectral collection system, **(B)** multispectral module with six specific bands.

As illustrated in Figure 1A, the M100 Quad-rotor UAV system (34) is adopted here for serving as a platform to carry the multispectral camera, considering its advantages of smooth reliability, flexibility and portability, and the reserved expansion interfacefor convenient hardware integration and secondary development. The ground workstation is responsible for information transmission, target identification, and system control.

### 3.2. The optimized MSS module

The multispectral sensing (MSS) module on a cruising UAV is used to acquire some specific spectral features of the target background for suspected human targets detection during the entire search process. In our study, a few specific multispectral bans are selected by observing and analyzing the sensitivity of different spectral bands to the environment and background. Consequently, some optimal bands would be picked out from numerous hyperspectral bands, which have the greatest ability to distinguish suspected human targets from the natural environment with complex background situations.

To pick out these discriminative spectral bands, a large number of preliminary measurement experiments were carried out to obtain the wavelength-relative reflectivity curve for green vegetation (to simulate background) and green camouflage (to simulate suspected injured human target outdoors) using a spectrometer. Just as what we analyzed and validate in our previous paper (32), six specific bands, including the blue band (450 ± 3 nm), green band (555 ± 3 nm), red band (660 ± 3 nm), red edge (710 ± 3 nm) and near-infrared (840 ± 3 nm and 940 ± 3 nm), were selected as spectrum components for custom multispectral cameras. According to the requirements above, the MS600 camera shown in Figure 1B was adopted in our study.

## 4. Cross-scene camouflaged human targets recognition method

### 4.1. Modeling analysis of cross-scene target recognition

The multispectral feature-based recognition method demonstrates good recognition performance in its own scenario, but the performance will be severely limited for a complex and dynamic ground-environment scenario. In order to clarify this phenomenon theoretically, we first model and analyze it.

**Figure 2:**
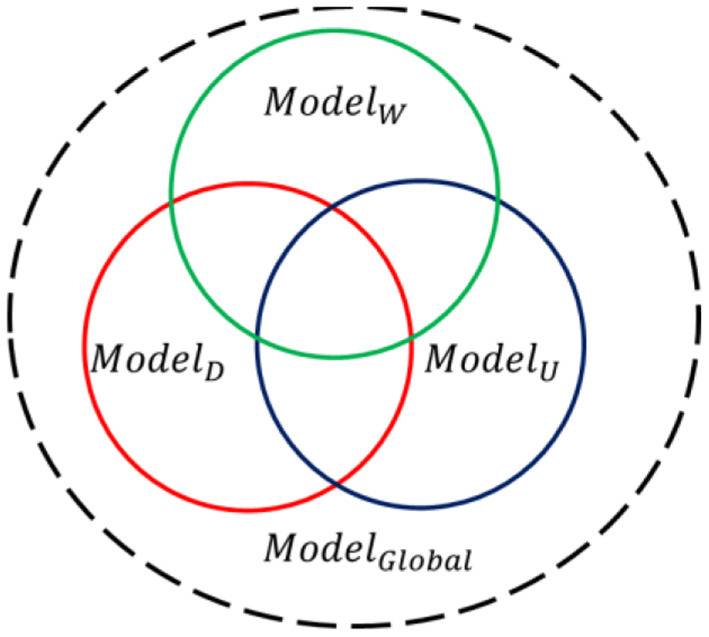
Schematic diagram of the relationship between different scene classification models.

In the practical application, the ground components in the environment are more complex and environment types are different and changeable during the searching process. Since the distribution location of the outdoor target is unknown and random, the ground environment in which the UAV system performs searching missions is also randomly unknown and even dynamically transformed (*Woodland, Desert* or *Urban*). However, it can be inferred from Figure 2 that the recognition model *Model*_*W*_trained based on woodland scene data could only get significantly lower accuracy for target recognition in desert scenes and urban scenes, showing poor multi-scene universality and robustness. At a deeper level, it is because although the set of spectral features used to describe the environmental background and target is fixed, the importance and contribution of the same feature element to the target recognition result varies greatly in different scenarios. That is to say, most of the features are environment sensitive, thus single-scene recognition model, namely the undermentioned single-scene multi-domain feature optimization model in “Section 5”, will inevitably undergo poor adaptability and unsatisfactory recognition performance in cross-environment situation. Therefore, it is of practical application to propose a global classification model *Model*_*Global*_ and could also maintain good classification performance simultaneously under dynamic environment scenario.

### 4.2. CMFJO method for cross-scene camouflaged human targets recognition

**Figure 3:**
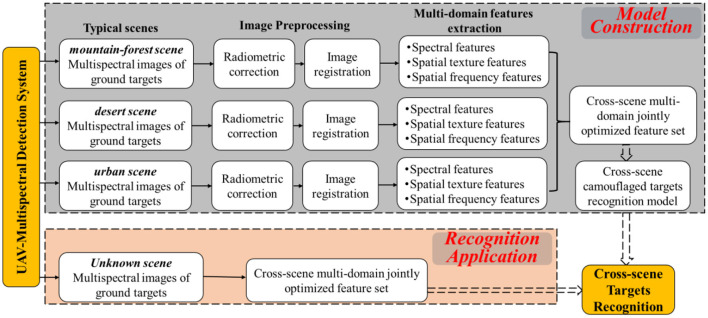
Scheme of cross-scene camouflaged human targets recognition.

Target to the aforementioned cross-scene recognition problem, the CMFJO method based on the UAV multispectral multi-domain features from multiple environmental scenes is proposed and its entire implementation process is illustriated by Figure 3. Next, the main steps of the CMFJO method and corresponding key methods are described in this section.

#### 4.2.1. The scheme of CMFJO method

##### 4.2.1.1. 1st step

Cross-scene multispectral images acquisition. By scanning ground targets based on the UAV multispectral camera system in the cruising state, six corresponding multispectral images can be acquired for each exposure. Depending on different scenes, cross-scene multispectral images of various ground targets could be collected. As for outdoor search applications, 3 typical ground scenarios are considered here, including desert, mountain forest, and urban scenes.

##### 4.2.1.2. 2nd step

Multispectral image preprocessing. As shown in Figure 3, the preprocessing on the above six single band images mainly includes two operations of radiation correction and band registration.

**Figure 4:**
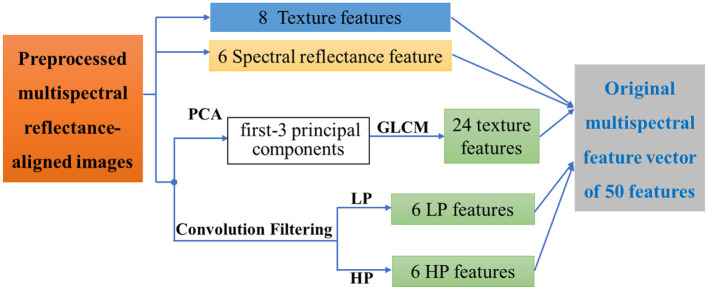
The diagram of multi-domain features from multispectral images.

1. Radiation correction. As shown in Figure 4, for the 6 single-band DN-value images acquired by the multispectral camera at each exposure, through identifying and calculating the average irradiance of the gray plate region based on the gray plate images, followed by the image aberration correction using downwelling light sensor, the original DN-value images would be correctly converted to reflectance images.
2. Waveband alignment. For a set of six reflectance images after radiometric calibration, direct image merging would cause serious pixel misalignment due to the obvious position offset between each band camera, which will bring about difference and ratio errors in the spectral index calculation. Therefore, high-precision band alignment would be conducted to eliminate such errors. Firstly, according to the geometric constraint relationship between optical lenses, Speeded Up Robust Features (SURF) algorithm is exploited to extract feature points. After feature point matching and affine transformation matrix calculating, the images are reprojected according to the affine transformation matrix, and then the corresponding six images are combined according to the band order, thus forming a 6-in-1 synthetic multispectral reflectance band aligned image.

##### 4.2.1.3. 3rd step

Multi-domain feature extraction. After dividing the ground targets into 2 major categories, namely human target and background, three-domain features including spectral features, texture features, and spatial frequency features, were extracted from the aligned 6-band images and the multispectral synthetic image, forming a multi-domain feature description set of the ground target.

Specifically, as shown in Figure 4, a total of 50 features of these types were extracted to form the multi-domain feature description set *F*_*Global*_:

(1) $$\begin{array}{l} \begin{array}{l} F_{Global}=\left\{\begin{array}{c} F_{reflect}\left(r_{b1},r_{b2},r_{b3},r_{b4},r_{b5},r_{b6},F_{index}\right)\\ F_{text}\left(Mean,Var,Con,Hom,Dis,Ent,ASM,Cor\right)\\ F_{conv}\left(hp_{b1},hp_{b2},\ldots ,hp_{b6};lp_{b1},lp_{b2},\ldots ,lp_{b6}\right) \end{array}\right\} \end{array} \end{array}$$

(2) $$\begin{array}{l} F_{index}={\;F}_{index}\left(NDVI,NDGI,NGBDI,PSRI,SIPI,mNDVI,MSR,EVI\right) \end{array}$$

1. **Spectral reflectance feature F**_**reflect**_: *r*_*b*1_ to *r*_*b*6_ are the radiation-corrected reflectance values corresponding to those six bands of multispectral images. *F*_*index*_ are eight spectral index features and their calculation methods have been talked about in our previous paper (32), which could enhance detail characteristics by combining the reflectance values of multiple bands.
2. **Texture features F**_**text**_: Texture is computed by the grayscale attribute of pixels and their neighbors, which helps to distinguish the phenomenon of “same-spectrum, different-spectrum”. Here the principal component analysis (PCA) is firstly used to downscale the 6 bands and only the first 3 principal components are retained, including Imag(*PCA*_1_), Imag(*PCA*_2_) and Imag(*PCA*_3_),. Thus each band corresponds to a set of 8 features, and then 24 texture features from *F*_*texture*_ (*PCA*_1_), *F*_*text*_ (*PCA*_2_), and *F*_*text*_ (*PCA*_3_) could be extracted using the grayscale co-generation matrix (GLCM) method, which can characterize the image grayscale direction, interval, change amplitude and speed, etc.
3. **Spatial frequency features F**_**conv**_: High-pass filtering and low-pass filtering are performed on the pre-processed six multispectral reflectance images. Consequently, *hp*_*b*1_ to *hp*_*b*6_ are the high-frequency features corresponding to those six bands, which correspond to the edge information between different regions. Meanwhile, *lp*_*b*1_ to *lp*_*b*6_ are the low-frequency features, which correspond to the low-frequency information of the image to obtain the grayscale changes and image details.

In summary, the multi-domain feature *F*_*global*_ can be expressed as:

(3) $$\begin{array}{l} \begin{array}{l} F_{Global}=\left\{\begin{array}{c} {F_{reflect},F_{index},F}_{text}\left(PCA_{1}\right),F_{text}\left(PCA_{2}\right),\\ F_{text}\left(PCA_{3}\right),F_{convo}\left(lp\right),F_{convo}\left(hp\right) \end{array}\right] \end{array} \end{array}$$

where *F*_*text*_ (*PCA*_*i*_) represents the grayscale co-occurrence matrix texture feature corresponding to the *i* − *th* principal component after PCA decomposition, *F*_*convo*_ (*lp*) and *F*_*convo*_ (*hp*) represent the low-pass and high-pass frequency features respectively. Finally, all the 50 features in 3 major domains are numbered to form a global feature vector *F*_*Global*_ shown in Equation (3).

##### 4.2.1.4. 4th step

Optimal feature vector selection from multi-scene multi-domain feature. Targeting the problem that the single-scene recognition model is inclined to expose poor recognition performance and weak robustness under switching scenes, here we try to filter out the superior feature subset from multi-domain features under multiple scenes, whose biggest advantage is that it can be adapted to ground human target recognition under various outdoor scenes, enhancing its recognition performance and practicality.

Firstly, three main types of outdoor ground environments (*Woodland, Desert* or *Urban*) are considered here, thus multi-domain feature description sets of different ground targets (camouflaged human target and ground background) in different scenes were obtained. Then, the SVM-based Recursive feature elimination (*SVM-RFE*) and *Relief* algorithms (*Both of them will be introduced in the following part*)are used to sort the original feature set according to the contribution of different features to the recognition result from largest to smallest, forming two sorting results *F*_*RFE*_ and *F*_*Reli*_. After that, taking the selected *Top-n* (1 ≤ n ≤ 50) features according to the ranking as input features, the SVM is exploited as a classifier to test the target recognition efficiency of the *Top-n* feature combination. In this way, multi-domain optimized feature vector, which can guarantee a good recognition performance in multiple scenarios with the smallest possible number of features, are automatically filtered.

Here, the classification accuracy (Acc) and the Area Under the Curve (AUC) of the Receiver Operating Characteristic (ROC) curve were calculated for each SVM model through 5-fold cross-validation, which aim to evaluate each model's classification performance. Considering the urgency of the UAV casualty search task and the fact that the experiments are pixel-level recognition with high error tolerance, the principle of selecting the smallest possible feature vector while avoiding significantly degrading the classification accuracy was adopted in the feature selection. Therefore, four feature vectors with local optimal performance could be obtained, including *MaxAcc*(*Top* − *k* − *F*_*RFE*_), *MaxAcc*(*Top* − *l* − *F*_*Reli*_), *MaxAUC*(*Top* − *m* − *F*_*RFE*_) and *MaxAUC*(*Top* − *n* − *F*_*Reli*_). Accordingly, four corresponding recognition models could be generated by training and then verified using test dataset. Finally, the classification result figure, key parameter table and ROC curves were exploited together to evaluate the classification performance, which helps filter out the optimal multi-domain feature vectors with stable and efficient target recognition capability for cross-scene situations F^*^:

(4) $$\begin{array}{l} \begin{array}{l} F^{*}=Max\{Confusion\;Matrix\\ \left\{\begin{array}{c} MaxAcc\left(Top-k-F_{RFE}\right),MaxAcc\left(Top-l-F_{Reli}\right),\\ \;MaxAUC\left(Top-m-F_{RFE}\right),MaxAUC\left(Top-m-F_{RFE}\right) \end{array}\right\}\} \end{array} \end{array}$$

##### 4.2.1.5. 5th step

Target classification. Based on sufficient feature datasets of different scenes, the recognition model (here the SVM is adopted) can be well trained and optimized. During the test stage, For a set of multispectral images with camouflaged human targets and environmental background detected by the UAV multispectral system in any scene, human targets can be recognized based on the above-mentioned merit feature vectors combined with the well-trained recognition model.

#### 4.2.2. SVM-RFE and relief algorithms for feature set optimization

##### 4.2.2.1. SVM-RFE-based feature set optimization

The SVM-RFE constructs the sort contention based on the weight vector W generated by the SVM during training. Each iteration removes a feature attribute with the smallest sorting coefficient, and finally the descending order of all feature attributes would be acquired.

The linear kernel function-*Relief* is adopted here:

(5) $$\begin{array}{l} \delta^{j}=\overset{n}{\underset{i}{\sum }}\left[-diff{\left(x_{i}^{j},x_{i,nh}^{j}\right)}^{2}+\overset{k}{\underset{l}{\sum }}P_{l}^{*}diff{\left(x_{i}^{j},x_{i,l,nm}^{j}\right)}^{2}\right]\; \end{array}$$

Where δ^*j*^ is related statistics of *j* attribute, $x_{i}^{j}$ represents the value of *j* attribute in sample *x*_*i*_, $x_{i,nh}^{j}$ is the value of *j* attribute of near-hit *x*_*i,nh*_ from the sample *x*_*i*_, *P*_*l*_ is the sample proportion of type *l*, $x_{i,l,nm}^{j}$ is the value of *j* attribute of near-miss *x*_*i,l,nm*_ from the sample *x*_*i*_, $diff\left(x_{i}^{j},x_{i,nh}^{j}\right)$ means the difference between *x*_*i*_ and *x*_*i,nh*_ in *j* attribute, and $diff\left(x_{i}^{j},x_{i,l,nm}^{j}\right)$ means the difference between *x*_*i*_ and *x*_*i,l,nm*_ in *j* attribute.

##### 4.2.2.2. Relief -based feature set optimization

Relying on the idea of “hypothesis margin,” *Relief* evaluates the classification ability of features on every dimension, so that the most useful feature subset for classification can be approximately estimated. The “hypothesis interval” refers to the maximum distance that the decision surface can move while keeping the sample classification unchanged, which can be expressed as :

(6) $$\begin{array}{l} \theta =\frac{1}{2}\left(\left||x-M\left(x\right)||-||x-H\left(x\right)|\right|\right) \end{array}$$

where *M*(*x*) and *H*(*x*) refer to the nearest neighbors that are homogeneous with *x* and that is not.

Supposing that the training set is *D* = {(*x*_1_, *y*_1_), (*x*_2_, *y*_2_), …, (*x*_*m*_, *y*_*m*_), for each sample *x*_*i*_, the near-heat *x*_*i,nh*_ could be acquired by calculate the nearest neighbor between *x*_*i*_ and the same-class sample. On the other hand, the near-miss *x*_*i,nm*_ coms from the nearest neighbor between *x*_*i*_ and the non-similar class sample. Consequently, the correlation statistic corresponding to attribute *j* is:

(7) $$\begin{array}{l} \delta^{j}=\underset{i}{\sum }-diff{\left(x_{i}^{j},x_{i,nh}^{j}\right)}^{2}+diff{\left(x_{i}^{j},x_{i,nm}^{j}\right)}^{2} \end{array}$$

where $x_{a}^{j}$ represents the value of *j* attribute in sample *x*_*a*_, and $diff\left(x_{a}^{j},x_{b}^{j}\right)$ depends on the type of attribute *j* (here is discrete attributes):

(8) $$\begin{array}{l} diff\left(x_{a}^{j},x_{b}^{j}\right)=\{\begin{array}{l} \;0,\;x_{a}^{j}=x_{b}^{j}\;\\ 1,\;otherwise \end{array} \end{array}$$

Through Equsation (7), the evaluation value of a single sample for each attribute can be obtained. By averaging all the evaluation values of all samples for the same attribute, the relevant statistical components of the attribute can be obtained, where the larger the component value means the stronger classification ability.

### 4.3. Experimental setup

**Figure 5:**
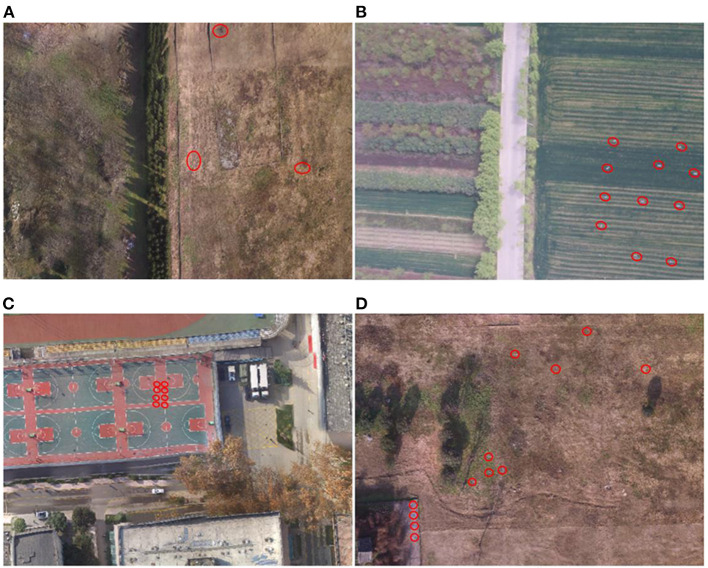
Experimental scenarios under different environments. **(A)** desert scenario; **(B)** woodland scenario; **(C)** urban scenario; **(D)** complex scenario.

Here, just as shown in Figure 5, we set up a dynamic cross-scene environment scenario set containing multiple typical ground environments (desert, woodland, and urban) and a complex scenario, so as to test the effectiveness and robustness of the proposed method for recognition under practical applications. The system parameters of the data collection system remain consistent, and only the ground environment scene changes. The uniform flight height was 100 m, and clear and breezy weather was selected. The air-to-ground detection data was acquired based on a small UAV multispectral system with a flight height setting of 100 m. Some key parameters were set as follows, aerial strip spacing of 27.4 m, the flight speed of 4.4 m/s, ground image resolution of 6.25 cm/pixel, and field of view angle width of 80 m^*^60 m. The automatic capture mode overlap trigger was used, with a heading overlap rate of 80% and a side overlap of 50%. In particular, a calibrated gray plate is taken before and after the flight for radiation correction. Simultaneously, different color camouflage uniforms were used to simulate injured human targets under corresponding experimental scenes, and their locations were randomly distributed. The main characteristic ground components in each experimental scenario are shown in Table 1.

**Table 1:** Main characteristic ground components of different experimental scenarios.

**Experimental scene**	**Main components**	**Simulated injured human targets**
Desert scene	Waste ground, shrubs, dead trees, dead grass, rocks	Desert camouflage
Woodland scene	Grass, trees, bare earth, weeds	Woodland camouflage
Urban scene	Buildings, roads, vehicles, vegetation	Urban camouflage
Complex scene	Buildings, tarmac, grass, vegetation Moorland, shrubs, dead grass, dead trees	Three kinds of camouflage

Based on the above uniform ground experimental setup, three typical outdoor scenes were selected for data acquisition. (1) For the desert scene, we selected the Han Chang'an City Ruins Park for similar desert scene data acquisition in December 2021 (winter), and could obtain 2,220 spectral images with different observation angles after multiple flight acquisitions. (2) For woodland scenes, 3 typical outdoor scenes were selected for data acquisition in the outskirts of Huxian, Shaanxi In June (summer), October (autumn) and March (spring) of 2020, a total of 2,214 multispectral remote sensing images of woodland scenes were collected several times. (3) For urban scenes, the stadium and surrounding buildings in the Fourth Military Medical University were selected as the background to simulate urban scenes for spectral data acquisition, and a total of 1,182 multispectral remote sensing images were obtained in December 2021.

Although a large number of spectral images from different observation angles are available for each scene, here only images from aerial orthogonal views are selected to maintain consistency in the impact parameters. For each image, multi-domain features are extracted to form the experimental data, and pure pixels are selected as experimental samples. Specifically, in the feature data acquisition stage of this study, the human target and background environmental components are first manually calibrated and segmented from the collected original data, then nd then the relevant spectral features are extracted automatically based our designed feature extraction algorithm. Since the feature species are fixed, the feature dimension is consistent.

In particular, after checking for sample integrity and removing outliers, the sample size is controlled by equally spaced sampling of the background samples to minimize the differences arising from the imbalance between positive and negative samples, so that the target and background sample sizes are close to a 1:1 ratio. In addition, we set the desert environment feature label as negative sample “0” and the camouflage target label as positive sample “1”. Finally, we obtain the number of environmental samples and target samples for different scenes. Further divided into training set and test set, we can obtain Desert dataset (3,665 environmental samples and 3,371 desert camouflage samples in the training set; 7,533 environmental feature samples and 7,184 desert camouflage samples in the test set), Woodland dataset (16,227 environmental samples and 13,187 woodland camouflage samples in the training set; 6,164 environmental feature samples and 5,502 woodland camouflage samples in the test set) and Urban dataset (10,568 environmental samples and 8,001 urban camouflage samples in the training set; 8,762 environmental feature samples and 7,700 woodland camouflage samples in the test set).

Based on the corresponding feature data sets of the above three typical scenes and an additional complex scene (shown in Figure 5D), the subsequent cross-scene recognition experiments based on the single-scene multi-domain feature optimization (SMFO) model and the cross-scene recognition experiments based on CMFJO model will be tested and analyzed.

### 4.4. SMFO model-based camouflaged target recognition under different single-scene environment

In general, the main process of training a model for target recognition from a single scene data consists of two main steps. First, multi-domain features are obtained for the background feature components and the simulated casualty target in the spectral image of the scene. Then, based on the feature selection method, the superior feature vectors for this scene are selected to build a recognition model based on the superior feature vectors to perform target recognition. The detailed process has already been explained in “Section 3.2” and will not be repeated here. Here we would like to explore and discuss the recognition effectiveness of the single-scene recognition model through multi-domain features optimization.

#### 4.4.1. Individual scene target recognition based on SMFO model

In order to investigate the recognition effect of a single scene recognition model in the case of feature selection, the multi-domain feature data set of multispectral remote sensing images based on any scene is divided into a training set and a test set. The SVM-RFE and Relief algorithms are then used to rank the features of the training set and reasonably select the superior feature vector to form the recognition model for target recognition in this scene.

##### 4.4.1.1. Desert scene experiment

**Figure 6:**
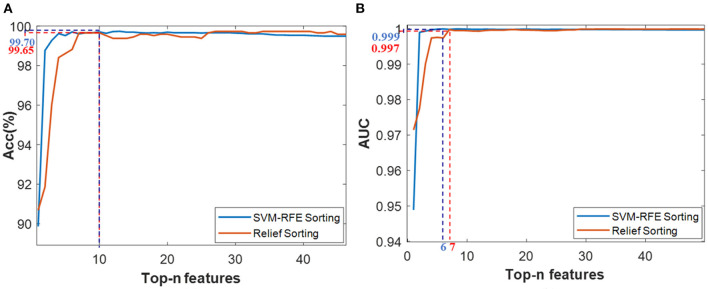
Classification results based on the Top-n features *via* two ranking methods of SVM-RFE and Relief, **(A)** Acc value, **(B)** AUC value.

Based on the desert scene dataset, five-fold cross-validation was performed after setting the SVM model parameters using the grid-seeking method. Then the average Acc and AUC results for each iteration were calculated separately as shown in Figure 6.

Considering the classification accuracy, the Top-10 features through RFE sorting (namely *Top-10-F*_*RFE*_) achieved a local optimum of 99.70%, while the top-10 features through Relief sorting (namely R_ *Top-10-F*_*Reli*_) also achieved a local optimum of 99.65%. Meanwhile, from the AUC results, the *Top-6-F*_*RFE*_ and *Top-7-F*_*Reli*_ achieved optimal classification performance of 0.999 and 0.997 respectively. Therefore, these four feature vectors were initially selected for the SVM recognition model training, thus forming four SVM models, whose recognition performance would be validated *via* test data set.

**Figure 7:**
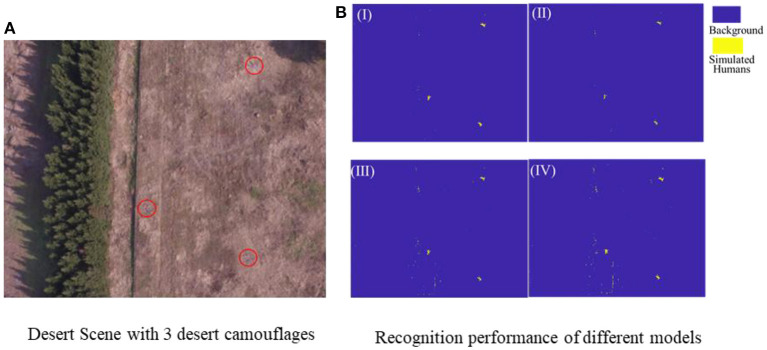
**(A)** Desert test scene with three desert camouflages, **(B)** Recognition results of different recognition models based on 4 superior feature vector, I (*Top-6-F*_*RFE*_), II(*Top-10-F*_*RFE*_), III (*Top-7-F*_*Reli*_), and IV(*Top-10-F*_*Reli*_).

During the desert scene test, as shown in Figure 7A, three desert camouflage suits were randomly laid on the ground to simulate battlefield casualties. Visually, the desert camouflage suits were so well camouflaged in the desert scenario that it was difficult to find the casualty target quickly through machine vision. However, just as shown in Figure 7B, the four recognition models recognized the three desert camouflage suits successfully based on the above 4 feature combinations screened out in the previous step. To further quantitatively evaluate the recognition results, a key parameter table was adopted for evaluation and the results are shown in Table 2. The results show that the *Top-6-F*_*RFE*_-based SVM model exhibits the best performance, thus it is chosen as the single-scene optimal feature vector for desert camouflage casualties searching in desert scenes, noted as $F_{D}^{*}$.

**Table 2:** Desert camouflage recognition results of 4 SVM models based on 4 superior feature vector in desert scene.

**Superior feature vectors**	**Accuracy**	**Sensitivity**	**Specificity**	**Precision**	**F1 score**	**Kappa**
*Top-6-F_*RFE*_*	**0.9635**	**0.9623**	**0.9648**	**0.9663**	**0.9643**	**0.9270**
*Top-10-F_*RFE*_*	0.9566	0.9527	0.9606	0.9621	0.9574	0.9131
*Top-7-F_*Reli*_*	0.9304	0.9060	0.9560	0.9557	0.9302	0.8609
*Top-10-F_*Reli*_*	0.9229	0.8979	0.9491	0.9487	0.9226	0.8459

##### 4.4.1.2. Woodland scenario experiment

**Figure 8:**
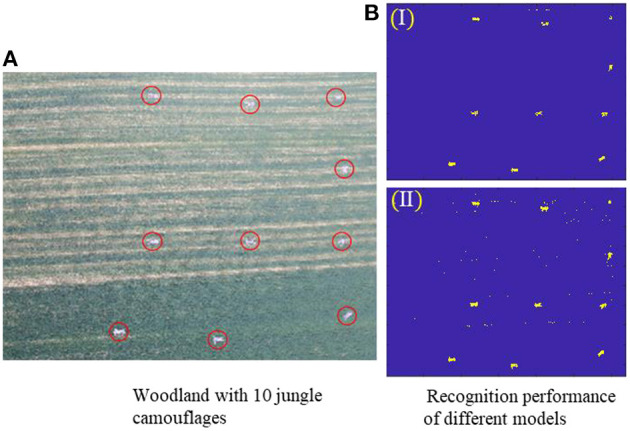
**(A)** Woodland test scene, **(B)** Recognition results of two recognition models based on two superior feature vector, I (*Top-10-F*_*RFE*_) and II (*Top-10-F*_*Reli*_).

The feature vector selection process for woodland scenes is similar to that for desert scenes. Based on the woodland scene dataset, two superior feature vectors *Top-10-F*_*RFE*_ and *Top-10-F*_*Reli*_were initially screened for validation. The woodland scenario test environment shown in Figure 8A, is mainly grass and a total of 10 jungle camouflage uniforms are laid to simulate battlefield casualty targets. From Figure 8B, the jungle camouflages could be recognized based on the 2 recognition models based on the *Top-10-F*_*RFE*_and *Top-10-F*_*Reli*_features vectors, respectively.

Just as classification results demonstrated by the key parameter table in Table 3, the *Top-10-F*_*RFE*_-based recognition model achieved better performance, thus it is chosen as the single-scene optimal feature vector for jungle camouflage casualties searching in woodland scene, noted as $F_{W}^{*}$.

**Table 3:** Jungle camouflage recognition results of 2 SVM models based on 2 superior feature vector in woodland scene.

**Superior feature vectors**	**Accuracy**	**Sensitivity**	**Specificity**	**Precision**	**F1 score**	**Kappa**
*Top-10-F_*RFE*_*	**0.9981**	0.9982	**0.9980**	**0.9982**	**0.9982**	**0.9962**
*Top-10-F_*Reli*_*	0.9962	**0.9995**	0.9925	0.9934	0.9964	0.9924

##### 4.4.1.3. Urban scenario experiment

Based on the urban scenes dataset, four feature vectors, including *Top-7-F*_*RFE*_ (with max Acc of 99.68%), *Top-10-F*_*Reli*_ (with max Acc of 99.74%), *Top-3-F*_*RFE*_ and *Top-7-F*_*Reli*_ (both with max AUC of 1), were initially selected as feature vectors to construct superior recognition model.

**Figure 9:**
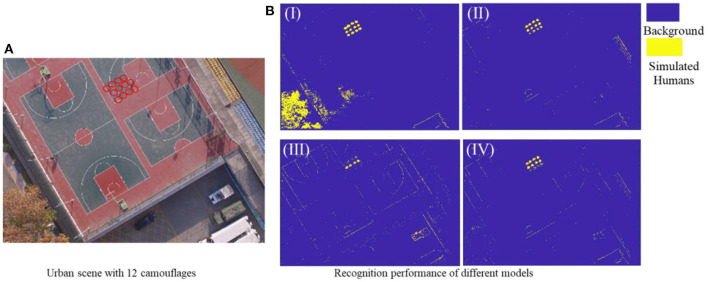
**(A)** Urban test scene with 12 camouflages, **(B)** Recognition results of different recognition models based on four superior feature vector, I (*Top-3-F*_*RFE*_), II(*Top-7-F*_*RFE*_), III (*Top-7-F*_*Reli*_), and IV(*Top-10-F*_*Reli*_).

As shown in Figure 9A, the urban scenario experimental environment contains a school basketball court and surrounding buildings, and 12 urban camouflages were laid centrally to simulate battlefield casualties. Then, these four superior models mentioned above were applied to recognize these urban camouflages under the urban environment. According to the classification results shown in Figure 9B and its quantitative key parameters in Table 4, the *Top-10-F*_*Relief*_-based recognition model achieved the optimum for all parameters, and thus was selected as the single-scene optimum feature vector for the camouflage casualties identifying in an urban environment, noted as $F_{U}^{*}$.

**Table 4:** Camouflage recognition results of 4 SVM models based on 4 superior feature vector in urban scene.

**Superior feature vectors**	**Accuracy**	**Sensitivity**	**Specificity**	**Precision**	**F1 score**	**Kappa**
*Top-3-F_*RFE*_*	0.8398	0.9069	0.7634	0.8135	0.8576	0.6756
*Top-7-F_*RFE*_*	0.9585	0.9906	0.9219	0.9352	0.9621	0.9163
*Top-7-F_*Reli*_*	0.9640	0.9642	0.9638	0.9680	0.9661	0.9277
*Top-10-F_*Reli*_*	**0.9739**	**0.9821**	**0.9647**	**0.9694**	**0.9757**	**0.9476**

#### 4.4.2. Cross-scene target recognition based on SMFO model

The aforementioned experiments revealed that each superior recognition model constructed from corresponding single-scene superior feature vector always exhibits an excellent recognition performance under its own scene. However, there is a fact that the searching ground environment is diverse and keeps switching dynamically and randomly. Therefore, it's necessary to investigate the recognition performance of a fixed-scene recognition model for other different types of scenes, like desert-scene model for woodland-scene recognition, or urban-scene model for woodland-scene recognition, etc.

**Figure 10:**
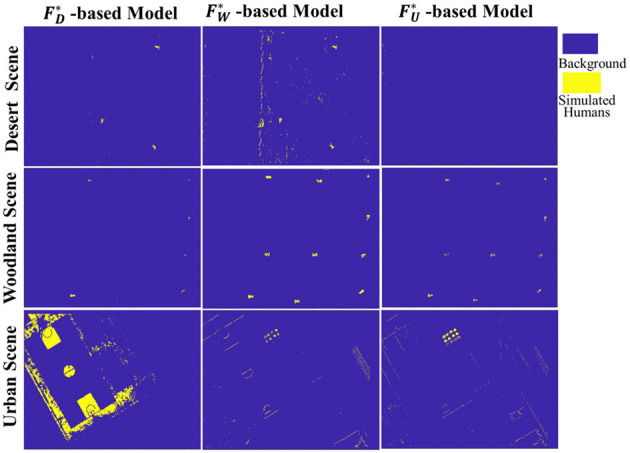
Cross-scene target recognition results based on SMFO model.

For this purpose, taking the SVM as the classifier, corresponding recognition models could be obtained based on the training data of different scenes, namely $F_{D}^{*}$ -based Model, $F_{W}^{*}$ -based Model and $F_{U}^{*}$ -based Model. Then, each SMFO model was adopted to classify the multispectral data of both its own scene and the other two scenes in turn and the results of scene cross-classification are shown in Figure 10. It can be intuitively seen that the recognition model of each typical scene has a good classification effect for unknown target data in the same type of scene. However, its recognition performance in other different scenes is unsatisfactory, indicating that the fixed scene recognition model has a large limitation and weak robustness.

### 4.5. CMFJO model-based camouflaged target recognition under cross-scene environment

To address the problem that the recognition performance of SMFO model deteriorates severely under cross-scene applications, the CMFJO model-based camouflaged target recognition method is proposed. It mixes all the feature training sets of three typical scenes together to form a comprehensive training set, and then a global optimal feature vector $F_{global}^{*}$ was filtered out to construct the CMFJO model, which could be well suited to cross-scene environment recognition in practical application. Meanwhile, as a reference method, we superimpose the superior feature subsets of those three typical scenes in *Part 4.2* directly and combine them to form a multi-scene combined feature vector $F_{glo}^{+}$, which is trained to construct a cross-scene recognition model based on the combined features of scenes.

#### 4.5.1. Reference recognition model

The superior feature subsets $F_{D}^{*}$, $F_{W,}^{*}$ and $F_{U}^{*}$ for the mentioned three typical scenes respectively have been selected. Then, they are directly superimposed to form the multi-scene combined feature vector $F_{glo}^{+}$, which are total of 18 features shown in Table 5 and were trained to construct a cross-scene recognition model.

**Table 5:** The overlay multi-scene combined feature vector $F_{glo}^{+}$ from 3 typical scenarios.

**No**.	**Parameter**	**Feature type**
4	Band 4 (710 nm)	Reflectivity
7	NDVI	Spectral index
8	NDGI	Spectral index
9	NGBDI	Spectral index
12	mNDVI	Spectral index
13	MSR	Spectral index
14	EVI	Spectral index
15	Mean of PCA_1_	Texture
17	Homogeneity of PCA_1_	Texture
20	Information entropy of PCA_1_	Texture
21	Second order matrix of PCA_1_	Texture
24	Variance of PCA_2_ PCA	Texture
27	Dissimilarity of PCA_2_	Texture
28	information entropy of PCA_2_	Texture
36	information entropy of PCA_3_	Texture
40	Low Pass Filter in Band 2 (555nm)	Spatial frequency
41	Low-pass filter in band 3 (360nm)	Spatial frequency
42	Low-pass filter in band 4 (710nm)	Spatial frequency

#### 4.5.2. CMFJO recognition model

**Figure 11:**
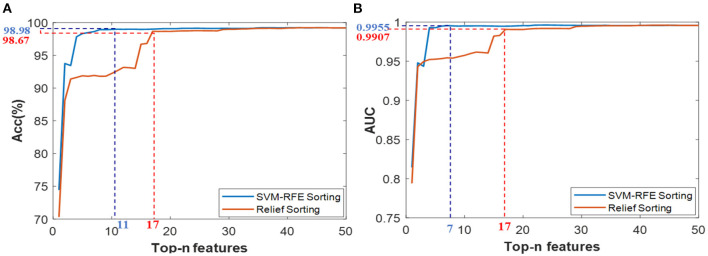
Classification results based on the Top-n features *via* two ranking methods of SVM-RFE and Relief under cross-cene situation, **(A)** ACC values of *Top-n* features from ensemble feature set, **(B)** AUC values of *Top-n* features from ensemble feature set.

Firstly, Based on the comprehensive training set consisting of all features of those three typical scenes, the SVM-RFE and Relief algorithms are exploited for feature ranking *via* five-fold cross-validation, and the results are shown in Figure 11. According to the results of Acc and AUC, the *Top-11-F*_*RFE*_ (local optimum Acc of 98.98%), *Top-17-F*_*Reli*_(with local optimum ACC of 98.67%), *Top-7-F*_*RFE*_(with local optimum AUC of 99.55%) and *Top-17-F*_*Reli*_(with local optimum AUC of 99.07%) were initially filtered out. Then, these three superior feature vectors and the multi-scene combined feature vector $F_{glo}^{+}$, were selected to conduct the target recognition experiments under multiple transformed scenes.

**Figure 12:**
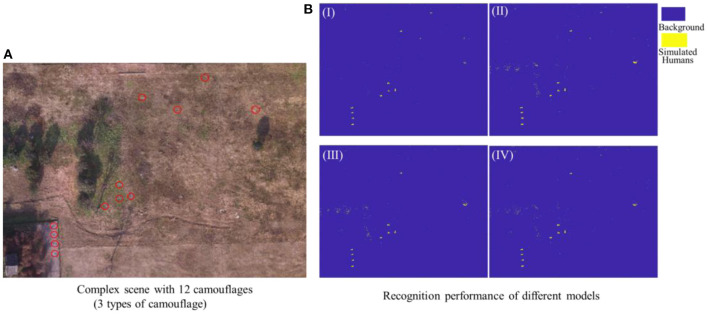
**(A)** Complex test scene, **(B)** Recognition results of different recognition models based on four feature vectors, I (*Top-7-F*_*RFE*_), II(*Top-11-F*_*RFE*_), III (*Top-17-F*_*Reli*_), and IV($F_{glo}^{+}$).

**Figure 13:**
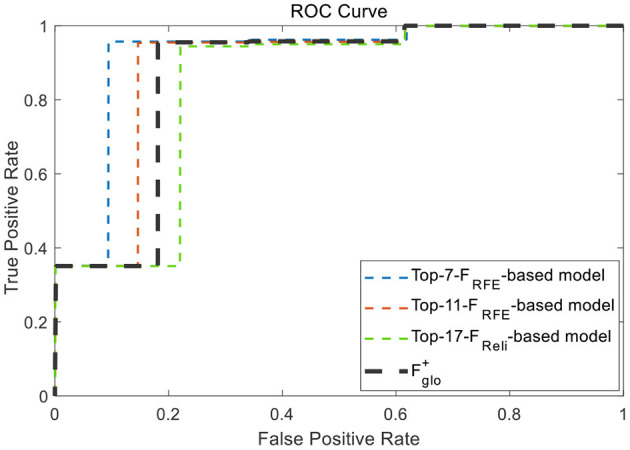
ROC curves of the four superior feature sets.

In order to test the recognition performance of the above four superior feature vectors under dynamic switching scenarios, namely cross-scene, a complex scene containing multiple scenes was deliberately set up as a test. As shown in Figure 12A, this scene contains most of characteristic components of three typical scenes, and meanwhile, each set of 4 camouflage uniforms (desert, woodland or urban) were used to simulate injured casualty targets lying flat position with casualty locations laid out randomly. The observational recognition results are shown in Figure 12 and its key parameter evaluation results are shown in Table 6. Further, the ROC curves of the four superior feature sets were analyzed and the results are shown in Figure 13.

**Table 6:** Complex scene classification results of four superior feature vector-based recognition models.

**Superior feature vectors**	**Accuracy**	**Sensitivity**	**Specificity**	**Precision**	**F1 score**	**Kappa**
*Top-7-F_*RFE*_*	**0.9255**	**0.8978**	**0.9574**	**0.9574**	**0.9266**	**0.8510**
*Top-11-F_*RFE*_*	0.8984	0.8485	0.9533	0.9524	0.8974	0.7974
*Top-17-F_*Reli*_*	0.8545	0.7746	0.9426	0.9369	0.8480	0.7109
$F_{glo}^{+}$	0.8807	0.8146	0.9536	0.9508	0.8775	0.7626

In summary, according to all the analysis above, it is clear that *Top-7-F*_*RFE*_holds the best recognition performance and can be taken as the cross-scene multi-domain optimal feature vector $F_{Cro}^{*}$, whose specific features are shown in Table 7.

**Table 7:** Cross-scene multi-domain superior feature vector $F_{global}^{*}$.

**No**.	**Parameter**	**Feature type**
42	Low pass filter in band 4 (710nm)	Spatial Frequency
40	Low pass filter in band 2 (555nm)	Spatial Frequency
4	Band 4 (710 nm)	Reflectivity
9	NGBDI	Spectral Index
20	information entropy of PCA1	Texture
46	High pass filter in band 2 (555nm)	Spatial Frequency
45	High pass filter in band 1 (450nm)	Spatial Frequency

#### 4.5.3. Multiple-scene recognition experiments

**Figure 14:**
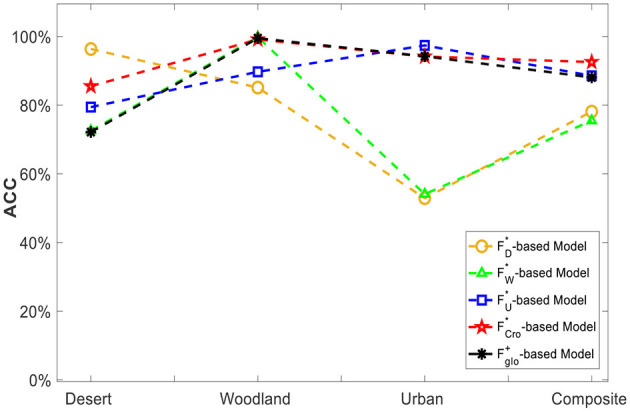
Classification ability comparison of single-scene merit feature set and cross-scene multi-domain superior feature vector.

Based on the aforementioned 5 feature vectors, including the single-scene optimal $F_{D}^{*}$, $F_{W,}^{*}$ and $F_{U}^{*}$, multi-scene combined feature vector $F_{glo}^{+}$ and the cross-scene optimal feature vector $F_{Cro}^{*}$, five corresponding recognition models were generated through training, respectively. Then, the testing datasets of *desert scene, woodland scene, urban scene* and c*omposite scene* were exploited to test the classification performance of each recognition model. As the classification results shown in Figure 14, it can be found that $F_{Cro}^{*}$-based model always maintains a high classification level of over 85% and always outperforms both the single-scene model and the combined-feature model. It strongly demonstrates that the selected optimal $F_{Cro}^{*}$ have a better characterization ability for different scenes, leading to its recognition model's environmental adaptation ability being significantly better than every SMFO model. Although the $F_{Cro}^{*}$-based model's performance in a few scenes is slightly worse than the signal-scene feature vector model $F_{D}^{*}$-based model for desert-scence recognition, $F_{U}^{*}$ -based model for urban-scence recognition, it's normal and is possibly related to the small volume of data.

The experimental results above clearly show that the SMFO models have serious limitations, and their classification performance is unsatisfactory in cross-scene conditions. On the contrary, the $F_{global}^{*}$-based CMFJO model acquired a classification accuracy of 92.55% in complex cross-scene conditions (desert, woodland, urban scene and composite scene), implying that it can improve the robustness and practical usability of the classification method for cross-scene situation while guaranteeing better performance. Therefore, the classification method proposed in this paper exhibit a promising recognition performance of outdoor camouflaged human targets using UAV multispectral system under low contrast environment.

## 5. Discussion

In view of the fact that the current ground human target recognition model is only oriented to a fixed single scene and cannot meet the cross-scene conditions in actual application, a CMFJO-based recognition method using UAV-mounted multispectral system is proposed. We can clearly see that the model trained under a single scene can effectively recognize targets in the same type of scene (desert scene with 96.35%, woodland scene with 99.81%, urban scene with 97.39%), but its recognition performance is severely degraded in other types of scenes. In fact, this is because the spectral, texture and spatial frequency characteristics of various ground components in a single type of scene have serious scene limitations and weak universality. Unfortunately, multiple types of scenes may occur in the practical cross-scenes condition and will contain various ground components, which means richer and more complex spatial distribution of these 3-domains multispectral parameter values. Consequently, the SMFO model will not be able to keep a good classification performance for such complex cross-scene multispectral feature data. Therefore, our proposed CMFJO method is just targeted to solve this problem, which can not only characterize objects from multiple aspects but also aims to enhance its cross-scene environmental applicability and cross-scene robustness.

Although the recognition is satisfactory to some extent for this challenging identification task, this method also has a few limitations for practical application in the future. Firstly, only three typical scenes were considered in this study while three are some other complex scenes in practical applications. Secondly, here we can just detect and recognize the camouflaged suspected human targets based on multispectral features. In the practical outdoor injured people search-and-rescue operation, what we need to do further is trying to realize the injured human attribution identification with the help of other additional sensing information, like morphological characteristics of the human body and even vital signs. Additionally, due to the feature extraction work in this study was carried out at the pixel level, so our dataset can meet the basic requirements of dataset size for the recognition experiments. But up to the image level for deep learning model-based human targets recognition study, our dataset is far from enough and more experiments in outdoor scenarios needed to be conducted to collect enough multispectral data.

## 6. Conclusions

In response to the challenging task of rapid search of ground injured human targets in outdoor environments, UVA-based multispectral detection and recognition technology is an effective but challenging new method and our previous research has preliminarily proved its feasibility.

Unfortunately, the human target exhibit low target-background contrast relative to the surrounding environment, and meanwhile the ground environment is variable and arbitrarily namely cross-scene transformed, so it is difficult to achieve stable and highly accurate target recognition under transformed scenes. Therefore, in this paper, we propose a CMFJO method for the cross-scene outdoor injured human target recognition.

In the experiments, we first screen the single-scene superior feature vector for each scene itself to construct the corresponding recognition model, namely the SMFO model, and then it was exploited to recognize targets in different scenes. Experimental results show that the SMFO model holds good recognition capability for its own scene (96.35% in desert scenes, 99.81% in woodland scenes, and 97.39% in urban scenes), but the recognition performance for other scenes deteriorates sharply (even below 75% overall) when the scene changes. In contrast, the CMFJO method proposed in this paper achieved an average classification accuracy of 92.55% in the cross-scene transformed situation. This result is very meaningful and important because it first time identifies the optimal multi-domain feature vectors with stable and efficient target recognition capability for cross-scene situations, and constructs a cross-scene superior recognition model. Therefore, it's promising to further enhance the accuracy and usability of UVA-based multispectral detection and recognition technology for outdoor injured human target searching in practical application conditions, like outdoor lost traveler search, casualty search in cross-domain combat and post-disaster casualty search. In the following work, we will try to exploite deep learning-based cross-scene recognition method into this research and to establish a large multi-scene outdoor human targets UAV-multispectral dataset (considering different human number, background, season, light and some other key factors).

## Data availability statement

The raw data supporting the conclusions of this article will be made available by the authors, without undue reservation.

## Ethics statement

Ethical review and approval was not required for the study on human participants in accordance with the local legislation and institutional requirements. The patients/participants provided their written informed consent to participate in this study.

## Author contributions

Conceptualization, supervision, and funding acquisition: GL and JW. Methodology: MZ. Software: ZL. Formal analysis: JX. Investigation: LZ. Data curation: YJ. Writing—original draft preparation and writing—review and editing: FQ. Visualization: MZ. All authors contributed to the article and approved the submitted version.
